# Intrinsic Determinants of A*β*_12–24_ pH-Dependent Self-Assembly Revealed by Combined Computational and Experimental Studies

**DOI:** 10.1371/journal.pone.0024329

**Published:** 2011-09-21

**Authors:** Weixin Xu, Ce Zhang, Philippe Derreumaux, Astrid Gräslund, Ludmilla Morozova-Roche, Yuguang Mu

**Affiliations:** 1 State Key Laboratory of Precision Spectroscopy, Institute of Theoretical and Computational Science, East China Normal University, Shanghai, China; 2 School of Biological Sciences, Nanyang Technological University, Singapore, Singapore; 3 Departments of Medical Biochemistry and Biophysics, Umeå University, Umeå, Sweden; 4 Laboratoire de Biochimie Théorique, UPR9080 CNRS, Institut de Biologie Physico-Chimique, Paris, France; 5 Department of Biophysics, Stockholm University, Stockholm, Sweden; University of Akron, United States of America

## Abstract

The propensity of amyloid-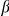 (A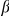) peptide to self-assemble into highly ordered amyloid structures lies at the core of their accumulation in the brain during Alzheimer's disease. By using all-atom explicit solvent replica exchange molecular dynamics simulations, we elucidated at the atomic level the intrinsic determinants of the pH-dependent dimerization of the central hydrophobic segment A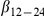 and related these with the propensity to form amyloid fibrils measured by experimental tools such as atomic force microscopy and fluorescence. The process of A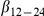 dimerization was evaluated in terms of free energy landscape, side-chain two-dimensional contact probability maps, 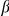-sheet registries, potential mean force as a function of inter-chain distances, secondary structure development and radial solvation distributions. We showed that dimerization is a key event in A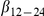 amyloid formation; it is highly prompted in the order of pH 5.0
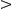
2.9
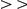
8.4 and determines further amyloid growth. The dimerization is governed by a dynamic interplay of hydrophobic, electrostatic and solvation interactions permitting some variability of 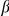-sheets at each pH. These results provide atomistic insight into the complex process of molecular recognition detrimental for amyloid growth and pave the way for better understanding of the molecular basis of amyloid diseases.

## Introduction

Alzheimer's disease (AD) or simply Alzheimer's is the most common form of dementia in aging people. The accumulation in the brain of amyloid deposits or plaques made of the amyloid 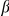-protein (A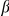) is a hallmark of AD. Although the toxic agents include soluble oligomers [1]–[4] as small as dimers [4], protofibrils [5]–[8] and mature fibrils [9], the etiology of amyloidoses is still poorly understood.

Knowledge of the polymerization at the molecular level, the structural details of fibrils, as well as the effects of external perturbations on fibrillation should facilitate the design of inhibitors. It is known that the predominant morphology is influenced by a variety of competing factors, including rates of spontaneous nucleation, fibril elongation, and fibril fragmentation. Stability of A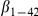 peptide fibrils is affected by environmental modifications, such as agitation [9], ionic strength [10], metal ions [11] and amino acid substitutions at various positions [12], [13]. The reproducibility of A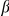 kinetics also depends on many factors including the monomer concentration at the start of each kinetic experiment [14]. The morphologies of the fibrillar structures and the aggregation kinetics are also highly sensitive to the pH values at which the protein solutions are incubated [15]. Clearly, we would like to gain insights into the effects of pH variations on the initial stages of A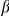 self-assembly at atomic resolution.

Structural characterization of these oligomers is difficult, however, by experimental means because they are transient in character. In addition, all-atom simulation study of the pH effect on A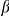 oligomers surrounded by waters is also intractable using current computer resources.

Here we focus on the studies of the early dimerization and subsequent fibrillar self-assembly of the A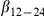 peptide, which corresponds to the central hydrophobic segment of the full length A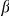 peptide, and therefore specific side-chain interactions and hydrogen bonding patterns of this peptide highlight the principles of the aggregation of the full length A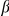. Indeed, ability to form amyloid fibrils of a range of A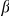 peptides fragments as well as inhibition of the full-length A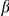 fibrillogenesis were examined in some previous studies and yet many questions regrading the nature of initial oligomers and specifically dimers remain open to debate. [16], [17]. The effects of pH on two A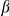 protein segments, A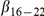 [18] and A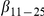, [19] have been characterized by solid-state NMR experiments. Both peptides form amyloids with antiparallel 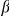-sheet geometries, but the H-bond register changes between A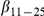 fibrils at pH 2.4, A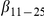 fibrils at pH 8.4 and A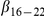 fibrils at pH 8.4. A recent computational study based on potential of mean force calculations along the distance separating the geometrical centers of 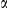 carbons located on two A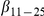 peptides that make up the two-stranded 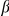-sheet was carried out [20]. It was shown that one registry is more stable at both neutral and low pH in the dimer, and the experimental difference in A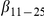 fibrillar registries is encoded at a higher level of organization. The main limitation of that study, however, is that the dimerization model was build not through self-assembly simulations; the number of the models obtained was thus limited and the ensemble statistical meaning was lost.

In contrast to A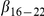 [21]–[23] and A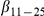 [20], [24], A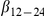 has never been the subject to any simulations, though it is known to form amyloid fibrils at pH 8.4 [25]. In addition to containing the central hydrophobic cluster (CHC) at positions 17–21, A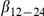 is also flanked at the N and C-termini by pairs of basic (His13, His14) and acidic (Glu22, Asp23) residues, i.e. residues that were suggested to act as gatekeepers in the fibrillation of other amyloids [26] when their charges are fully turned on. Evidently the gatekeeping function is pH-dependent. It is interesting to note that many mutations leading to familial AD are clustered at positions 22 and 23: Arctic (E22G), Dutch (E22Q), Italian (E22K), and Iowa (D23N).

To gain insights into the effects of pH on the early events of A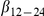 peptide self-assembly, we used replica exchange molecular dynamics (REMD) simulations of the dimers in explicit solvent at three pH values: 2.9, 5.0 and 8.4 and correlated the early dimerization steps with the fibrillation of A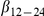 monitored by thioflavin T binding amyloid assay and AFM. We have shown that the modulation of pH has a very profound effect on both the early and late assembly process of the A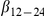 peptide and the early dimerization is crucial for the subsequent fibrillation events.

## Materials and Methods

### Experimental setups

The sequence of the A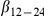 peptide is VHHQKLVFFAEDV; highly pure A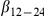 was produced by chemical synthesis. All experiments were performed with peptide weight concentration of 1 mg/ml determined by weight and Bradford assay under non-fibrillation condition (basic pH). In order to insure the disaggregation of the peptide prior the measurements we followed the protocol outlined previously [27]. The chilled powdered peptide was initially dissolved in 10 mM NaOH just above 1 mg/ml concentration, sonicated in an ice cold water bath for 1 min, and then ca. 10–20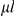 of 1 M NaH2PO4 buffer was added to adjust the environmental condition to a final buffer concentration of 20 mM and corresponding pH: 2.9; 5.0 and 8.4; after dilution pH was controlled by pH meter.

ThT binding assay was performed at 296 K as described in our previous publication [28]. Fluorescence of ThT was measured on a FP-6500 spectrofluorometer (Jasco) using excitation at 440 nm, emission at 485 nm, and setting the excitation and emission slits at 5 nm. The relative values of ThT fluorescence intensity were presented after being normalized on the fluorescence of free dye in solution.

Atomic Force Microscopy (AFM) measurements were performed on a PicoPlus AFM (Agilent) in a tapping mode using a 100 nm scanner under ambient conditions. Acoustically driven cantilevers had etched silicon probes of the TESP model with diameters of 10 nm and less (Digital Instruments). We applied a resonance frequency in the range of 170 and 190 kHz, a scan rate of 1 Hz or less, and a resolution of 512×512 pixels. Height, amplitude and phase data were collected simultaneously in trace and retrace to avoid the scan artifacts. Amyloid samples were deposited on the surface of freshly cleaved mica (GoodFellow) for 5 minutes, 3× washed with ca. 200 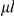 of MilliQ water and dried in air at room temperature. The dimensions of amyloid species were measured by multiple cross sections in AFM height images using PicoPlus software (Agilent).

### Replica Exchange Molecular Dynamics Simulations

Replica exchange method is a highly efficient sampling technique which was first implemented in molecular dynamics simulations by Hansmann [29], Sugita and Okamoto [30] and is widely used in studying protein folding and aggregation [31]–[41]. In replica exchange molecular dynamics (REMD) simulations, N non-interacting replicas at N different temperatures are conducted simultaneously in parallel. After a certain MD time, exchanges between neighboring replicas 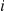 and 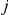 are attempted and accepted according to the Metropolis criterion: : 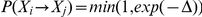, where 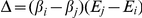 with 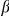 and E being the invert temperature and potential energy, respectively. With this method, the configurations at low temperatures can overcome high-energy barriers by being switched to high temperatures, and the resulting enhanced sampling gives a better description of thermodynamics at lower temperatures than standard MD.

### Simulation Protocol and Analysis

The peptide was capped by N-terminal acetyl (ACE) and C-terminal N-Me amide (NME) groups. To mimic different experimental pH values, the side chains of the residues His13, His14, Glu22 and Asp23 were modeled to take different charge states as shown in Table 1.

**Table 1:** Charge states for the His, Asp and Glu residues under different pH values.

		Charge State		
pH	HIS	GLU	ASP	LYS
2.9	+1	0	0	+1
5.0	+1	−1	−1	+1
8.4	0	−1	−1	+1

The simulation system is composed of two A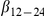 peptides that were represented by all-atom Amber03d force field [42] and solvated in a dodecahedron box of TIP3P waters. Totally three REMD simulations were performed at three pH values: (a) pH 2.9; (b) pH 5.0 and (c) pH 8.4, respectively. The initial conformation of the two peptides were fully extended and the inter-peptide distance was 2 nm. Both parallel and antiparallel geometries in equal amount of the two peptides were considered as starting structures to accelerate the convergence of REMD and avoid any bias [39]. The final setup of the system contained 3122 TIP3P water molecules and 6 CL- (chloride ions) at pH 2.9; 3126 TIP3P water molecules and 2 CL- at pH 5; and 3129 water molecules and 2 NA+ (sodium ions) at pH 8.4.

The GROMACS program suite version 3.3.3 [43] was used. All bonds involving hydrogen atoms were constrained in length according to LINCS protocol [44]. Electrostatic interactions were treated with the particle mesh Ewald method with a cutoff of 0.9 nm, and a cutoff of 1.4 nm was used in the calculation of van der Waals interactions. The integration time step was set to 0.002 ps. The peptides and the water groups were separately coupled to an external heat bath with a relaxation time of 0.1 ps using Berendsen coupling scheme [45]. Pressure coupling is switched off by fixing the volume. Non-bonded pair lists were updated every 0.01 ps. After 1000 steps of steepest-descent minimization, the REMD simulations were launched for 200 ns. The number of replicas is 64, and the temperatures were varied from 315.0 K to 513.5 K using the method implemented by Patriksson and van der Spoel [46]. The 64 temperatures, 315.00, 317.53, 320.09, 322.66, 325.25, 327.85, 330.47, 333.11, 335.77, 338.44, 341.14, 343.85, 346.58, 349.33, 352.09, 354.88, 357.69, 360.51, 363.34, 366.20, 369.08, 371.98, 374.91, 377.85, 380.82, 383.80, 386.80, 389.82, 392.87, 395.93, 399.02, 402.12, 405.25, 408.40, 411.57, 414.77, 417.98, 421.22, 424.47, 427.75, 431.05, 434.38, 437.72, 441.09, 444.49, 447.90, 451.34, 454.81, 458.30, 461.81, 465.34, 468.90, 472.48, 476.09, 479.72, 483.38, 487.06, 490.77, 494.50, 498.25, 502.04, 505.85, 509.68, 513.54, result in approximately 30% averaged acceptance ratio, with exchanges between neighboring replicas tried every 2 ps. The conformation coordinates were saved every 1 ps. After 200 ns, each REMD generated an ensemble of 200,000 structures at each temperature and totally 12,800,000 structures at all temperatures. The cumulative simulation time is 38.4 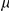
s. All time-averaged results presented below are based on the last 150 ns simulation data at the lowest temperature T 315 K, i.e. near the physiological temperature. The statistical errors were obtained from block averaging through dividing the last 150 ns data into 5 equal segments.

The DSSP algorithm was used to identify secondary structure conformation of the dimers [47]. A modified principal component analysis (PCA) method, referred to as dihedral angle PCA or dPCA, was used to construct the free energy landscape [48]. The absolute entropy was estimated by the quasiharmonic analysis or the essential dynamics method [49]. The covariance matrix is constructed as,
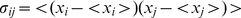
(1)where 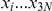 are the mass-weighted Cartesian coordinates of the N-particle system and 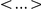 denotes the average over all sampled conformations. Hence, the covariance matrix provides information on the correlated fluctuations of pairs of atoms. The eigenvectors and eigenvalues 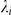 of 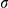 yield the modes of collective motion and their amplitudes. Based on the quasiharmonic approximation the configuration entropy is given by
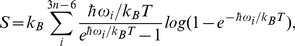
(2)where 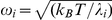, 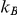, 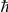 and 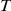 are the Boltzmann constant, reduced Planck constant and the temperature, respectively.

For further analysis, a single neutral or charged ASP/GLU/HIS residue was solvated in a cubic box with length of 2 nm. The N and C-termini of each residue were protected by ACE and NME, respectively. MD simulations were run for 10 ns with the temperature kept constant at 315 K.

## Results

### Experimental observations on A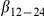 aggregation

**Figure 1:**
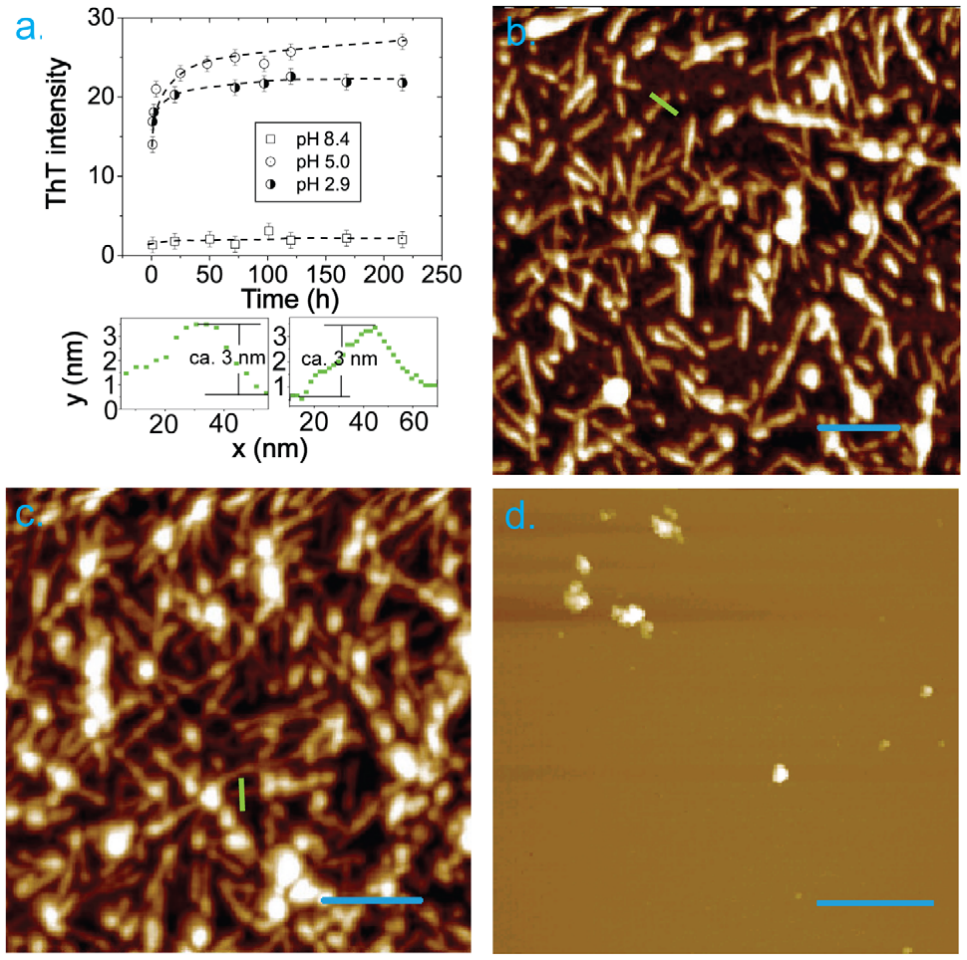
Kinetics of A 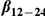
 **amyloid formation monitored by ThT fluorescence assay at 23** 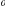 **(296 K) and pH 2.9 (black line and symbols); pH 5.0 (blue) and pH 8.4 (red) (a, upper panel).** The cross-sectional dimensions of the fibrils(a, lower panels). The position of cross-sections are shown by light green lines in (b,c); the left image corresponds to the fibril selected in (b) and the right one - in (c), respectively. AFM images of A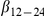 amyloids formed at pH 2.9 (b), pH 5.0 (c) and pH 8.4 (d) after 2 days of incubation. The scale bars represent 200 nm in (b),(c) and (d).

The aggregation behavior of A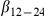 at the three pH values was characterized by ThT binding fluorescence assay and high resolution AFM imaging. The kinetics of amyloid formation was monitored by ThT binding as the specific interaction of ThT dye with cross-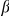 sheet containing amyloids leads to an increase of its fluorescence emission (Fig. 1a, upper panel). The time dependences of amyloid formation of ca. 1 mg/ml 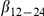 in 10 mM NaOH/NaH2PO4 at 296 K, pH 2.9 and 5.0 were marked by very fast growth phases characterized by a steep increase in fluorescence and ending at stationary phases after ca. 2 days, at which time thioflavin T fluorescence reached a plateau. The initial amyloid self-assembly proceeded so rapidly that the lag-phases could not be detected at both pH values. The plateau levels of relative fluorescence intensity of ThT bound to A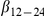 amyloids were by ca. 22 and 25 fold higher at pH 2.9 and 5.0, respectively, than that of free ThT in solution, reflecting significant amount of 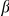-sheet formed at both pH and in particular at pH 5.0. By contrast, we observed a slight increase by ca. 2 fold of ThT florescence during incubation of A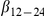 at pH 8.4, indicating that only a small amount of amyloids developed under this condition. We also performed ThT florescence measurement at pH 7.4 and found the signals are undistinguishable from those at pH 8.4.

Similarly, by AFM imaging we observed massive amyloid fibrils of A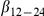 at both pH 2.9 and 5.0 after 2 days of incubation (Fig. 1b and c), but only unstructured aggregates were developed at pH 8.4 (Fig. 1d). The latter does not exclude the formation of small amount of amyloid fibrils at pH 8.4, but they were clearly not dominant species compared to the amyloids formed at lower pH, though reversely at lower pH we observed also some unstructured aggregated clumps (Fig. 1b and c). The fibrils at pH 2.8 were from hundred to a few hundred nanometer long and at pH 5.0 they grew even longer reaching a micron length. The height of individual fibrils at both pH values was ca. 3 nm as measured in AFM cross-sections (Fig. 1a lower panels), however at pH 5.0 there were more fibrils which were inter-wounded with each other forming thicker bundles (Fig. 1c). In both samples the fibrillar structures were rather straight and rigid and the lack of their flexibility was particularly evident in the pH 2.9 sample containing shorter fibrils. In summary, based on the results of both ThT binding assay and AFM imaging we can conclude that pH 5.0 is the most favorable for amyloid formation of 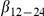 among three considered pH conditions.

### Simulation Results

#### Convergence evaluation

The goal of the simulation is to explain the observed pH effect on A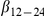 fibril formation by determining the structures and energetics of the dimer. We first checked the convergence of the simulations which is critical to the validness of results obtained. To check sampling efficiency and convergence degree, we followed the history of temperature swapping of each replica, and the time evolutions of the conformational entropy, S, (in the form of -T*S) [50], [51] and the averaged 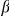-sheet lengths at 315 K.

**Figure 2:**
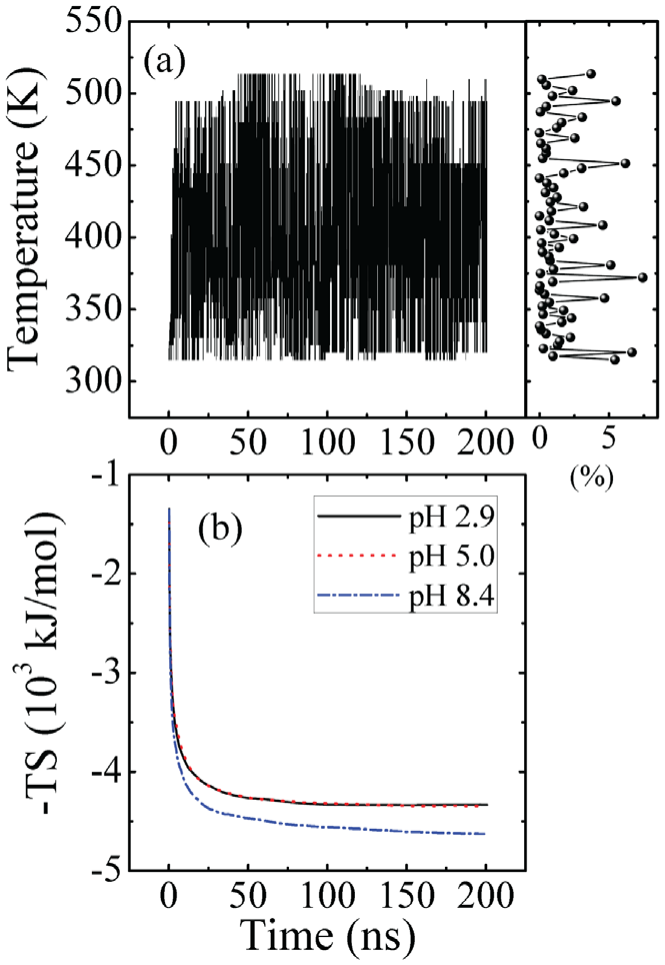
Evolution of temperature swapping of one replica at pH 2.9 (a, left panel). Percentage of simulation time dwelled in each temperature ladder (a, right panel). Time evolution of the conformational entropy at 315 K (b).

Fig. 2a (right panel) shows the walk of one replica along the ladder of temperatures as a function of time at pH 2.9. One can see that this replica explores the full spectrum of temperatures. The percentage of dwell time at each temperature is also shown in Fig. 2a (right panel). Ideally the percentage of dwell time at each temperature for a system of 64 replicas should be 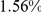. Here we find this replica stays in 9 temperatures with populations between 5% and 7.6% and these temperatures are nearly equally distributed within the 315–513 K range. Other replicas (including those at different pH values) show similar behaviors (data not shown).

The time evolution of the conformational entropy at 315 K is displayed in Fig. 2b. The entropy becomes nearly constant after about 60 ns at pH 2.9 and 5.0. At pH 8.4, however, the entropy still increases little even at simulation time of 200 ns, indicating a much larger conformational heterogeneity of the peptide than at the lower pH values.

**Figure 3:**
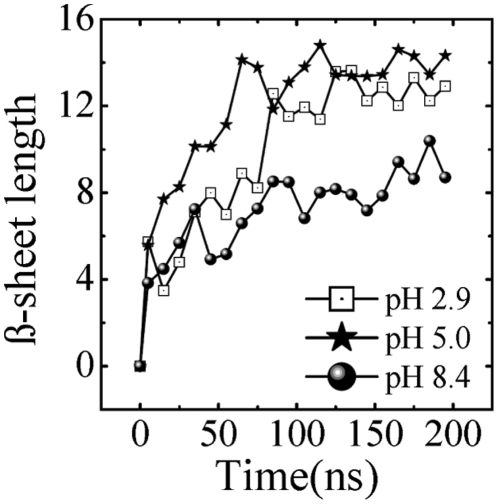
Time evolution at 315 K of the average 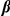
 **-sheet length, which is the number of residues involved in the** 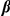 **-sheet structure.**

Figure 3 shows the time evolution of the total 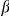-sheet length calculated by the DSSP algorithm [47]. Here the 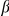-sheet length is the number of residues forming a continuous stretch with hydrogen bonding interactions. At 315 K, the amount of 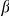-sheet formed is clearly different for the three pH values. In the beginning, there are no 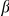 structures. The 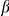-sheet structure accumulates with time, until around 100 ns, where a plateau is reached. This converged 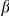-sheet length holds even at pH 8.4, suggesting that if completeness of the phase space is not reached considering the conformational entropy, the sampling is rather satisfactory and will not affect much our qualitative results at this pH. At 200 ns the 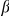-sheet length is about 13, 14.3 and 9 at pH 2.9, 5.0 and 8.4, respectively and thus the smallest length is observed at pH 8.4.

#### pH effects on free energy landscapes

**Figure 4:**
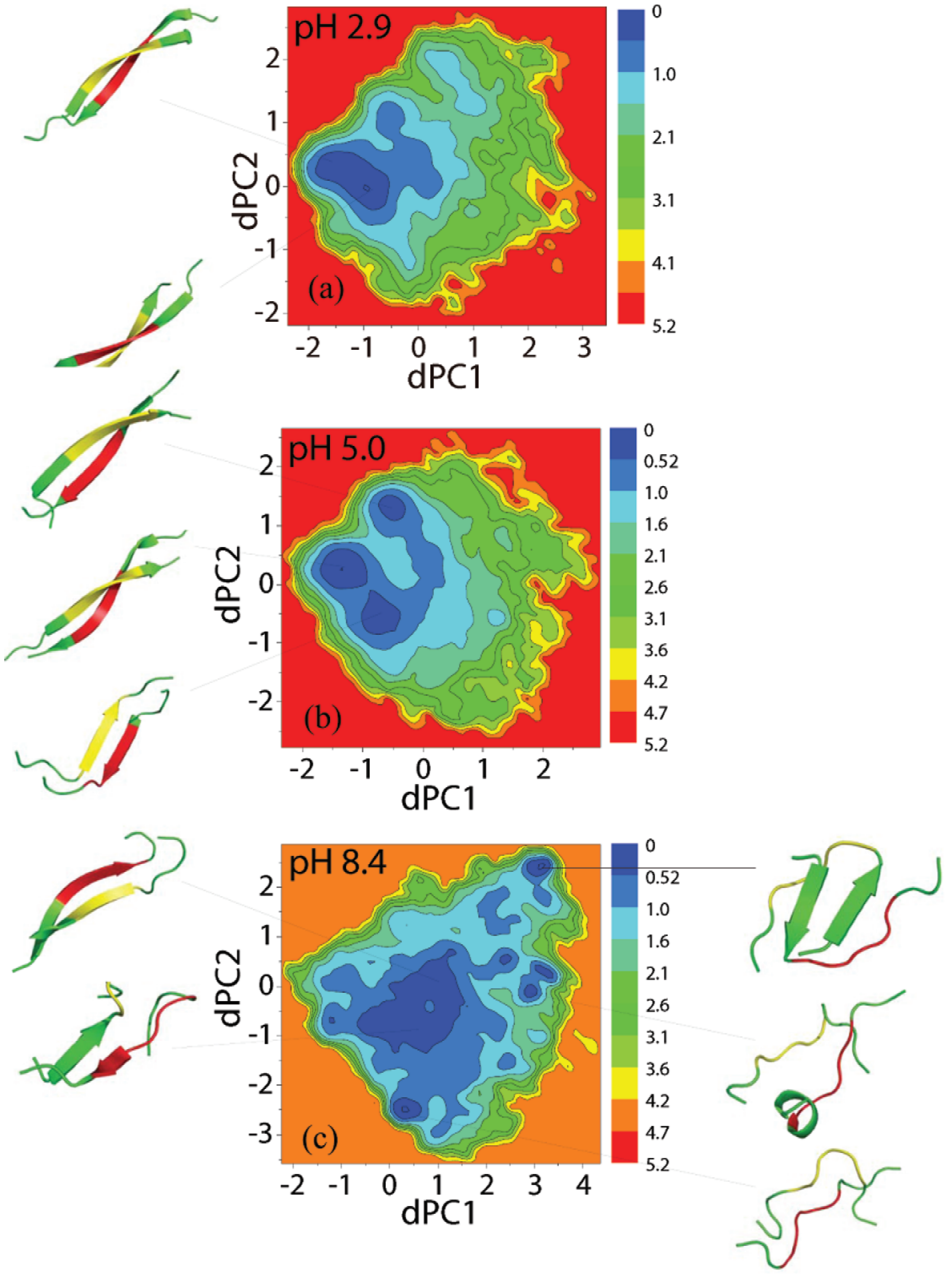
Free energy landscapes (in kJ/mol) as function of the first two dihedral principal component dPC1 and dPC2 at different pH values of 2.9, 5.0 and 8.4 at 315 K. The representative structures associated with the dominant minima are plotted by the PyMOL package [52]. The hydrophobic patch spanning LEU17-ALA21 is colored as yellow and red in each peptide. The statistical errors are smaller than 1 kJ/mol.

The free energy landscapes shown in Fig. 4 were constructed based on a dihedral angle principal component analysis (dPCA) method [48]. Using the first two principal components, dPC1 and dPC2, the multiple minima feature of the free energy surfaces is evident. For each local minimum, we performed the clustering analysis based on pair-wise RMSD structural comparison, and extracted the representative structures shown as snapshots drawn by PyMOL [52]. The populations of these structures are also given. Comparing the three free energy surfaces at 315 K, there are more minima at pH 8.4 (with many states within 1 kJ/mol) than at lower pHs where the minima are more clustered around specific dPC1 and dPC2 regions. The dominant structures of the pH 8.4 ensemble consist of both random coil structures and short antiparallel 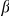-sheet structures (Fig. 4a). The dominant structures at pH 5.0 (Fig. 4b) and pH 2.9 (Fig. 4c) consist of more ordered 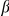-sheet motifs.

**Figure 5 pone-0024329-g005:**
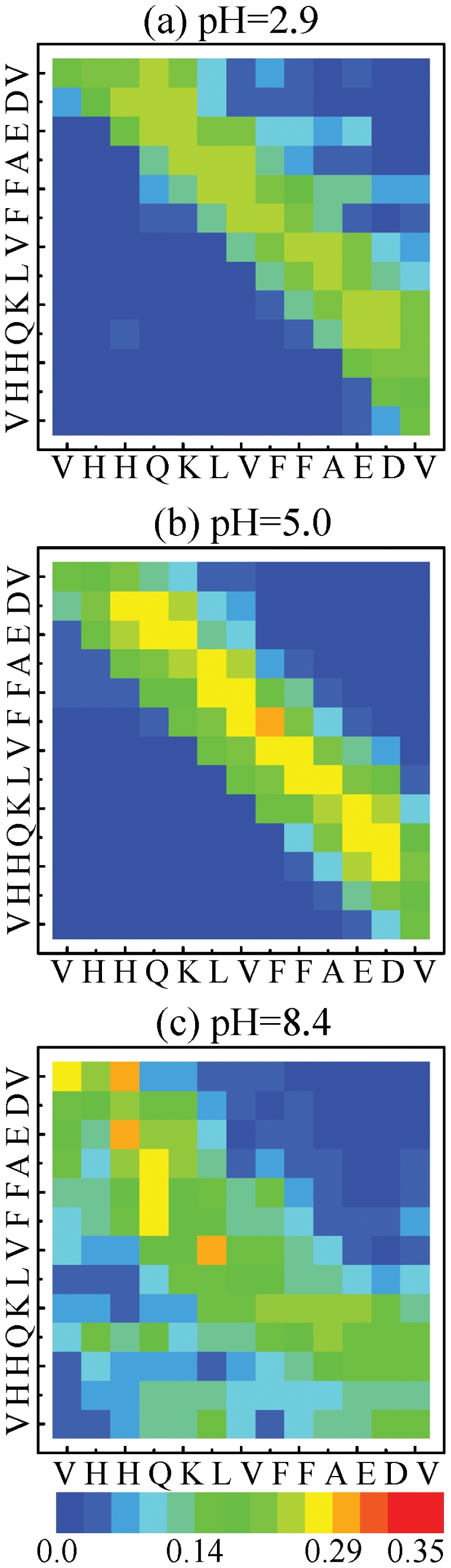
Side-chain – Side chain contact probability maps at 315 K using all conformations at the three pH values.

Further insights into the equilibrium structures at 315 K can be provided by the side-chain (Sc) – side-chain (Sc) contact probability maps using all sampled configurations. Consistent with the conformational entropy analysis, the 2D map at pH 8.4 does not show a well defined interpeptide interaction pattern, although there is a probability of 0.25–0.30 for scattered interactions between His14-Glu22 and Leu17-Val18 interactions (Figure 5c). Given that His14 is not charged at pH 8.4, these interactions can be attributed to non-specific hydrophobic contacts between the side chains of the above residues This runs strongly in contrast with the 2D map at pH 5.0 where we observe high probability of contacts within nearly the whole range of the peptide and notably between the two Phe19 residues(Fig. 5b). Clearly the 2D map at pH 2.9 in Fig. 5a is also much more organized than that at pH 8.4, but is less uniform and displays lower probabilities than what is observed at pH 5.0.

#### Parallel and antiparallel 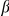-sheet patterns

**Figure 6 pone-0024329-g006:**
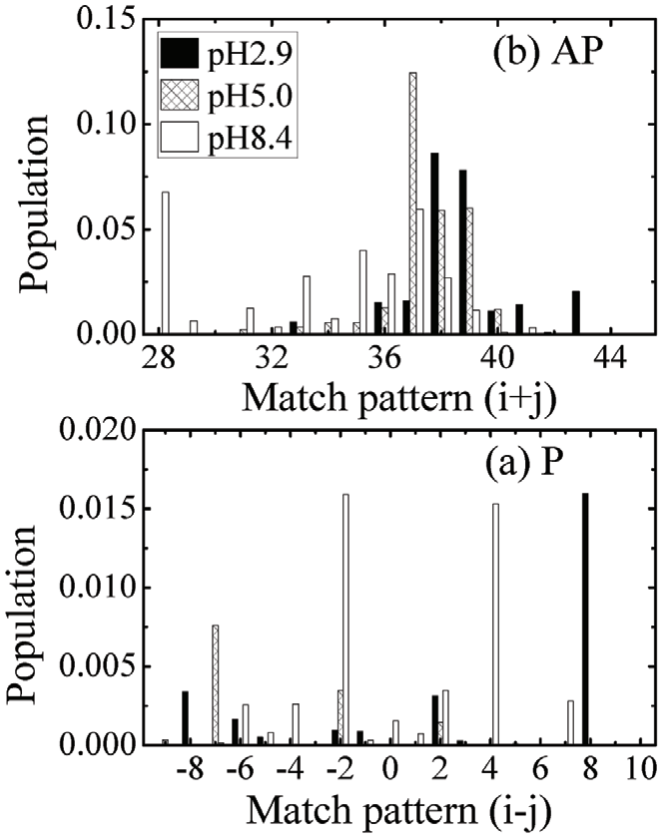
Population of various residue matching patterns for the parallel(P) (a) and antiparallel(AP) (b) alignments. For parallel alignment the difference of two hydrogen-bonded residue numbers (i−j) is the X-axis label; for antiparallel alignment, the sum of two hydrogen-bonded residue numbers (i+j) is the X-axis label. The statistical errors are smaller than 0.03.

**Figure 7:**
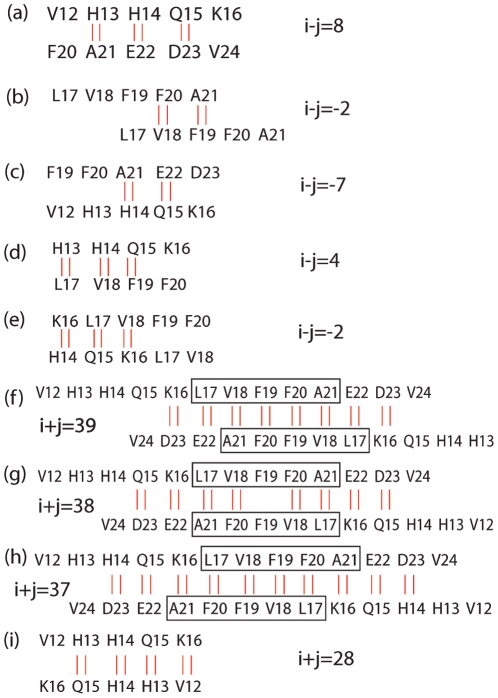
The highly populated parallel and antiparallel dimerization patterns of A 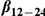
 **peptides at different pH levels.** Here two short red vertical lines denote one hydrogen bond. The boxes highlight the CHC regions.

Using all sampled configurations, Figure 6 gives the probability of each 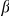-sheet pattern with parallel (Fig. 6a) and antiparallel (Fig. 6b) orientations at the three pH values. To distinguish the 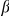-sheet registries, the difference of two paired residue numbers, i−j, denotes the parallel patterns in Fig. 6a; while the sum of two paired residue numbers, i+j, characterizes the antiparallel patterns in Fig. 6b. The corresponding residue-based alignments are illustrated in Fig. 7.

For the parallel 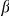-sheets, the following patterns are observed: (1) at pH 2.9, i−j = 8 (Fig. 7a), (2) at pH 5.0, both i−j = −2 and i−j = −7 shown in Fig. 7b and 7c, and (3) at pH 8.4, both i−j = 4 and i−j = −2 shown in Fig. 7d and 7e along with many others. It is to be noted, however, that all parallel patterns have a small probability of occurrence (P
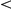
0.02) and their probabilities are within statistical errors.

For the antiparallel 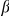-sheets: (1) at pH 2.9, the i+j = 39 (Fig. 7f) and i+j = 38 (Fig. 7g) patterns are highly populated (each with 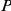 = 0.07), (2) at pH 5.0, the i+j = 37 (Fig. 7h) is dominant (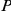 = 0.12), followed by the i+j = 38 and 39 patterns, each with 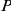 = 0.06 and (3) at pH 8.4, the i+j = 28 (Fig. 7i) and i+j = 37 (Fig. 7h) patterns are almost equiprobable (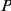 = 0.06).

Such a diversity of populated 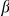-sheet registries has also already been observed by computational studies on other A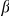 fragments; for instance in small oligomers of A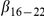 using either the OPEP coarse-grained force field [21], [53] or an all-atom model with implicit solvent [54], [55], and tetramers of A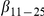 [24]. Interestingly, the i+j = 39 pattern shown on Fig. 7f matches the NMR 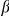-sheet registry of A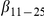 fibrils at pH 2.4 [18], the i+j = 38 pattern shown on Fig. 7g matches the NMR 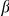-sheet registry of A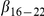 fibrils at pH 8.4 [19], and the i+j = 37 pattern shown on Fig. 7h matches the NMR 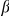-sheet registry of A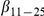 fibrils at pH 8.4 [18]. Of interest also is that the i+j = 37 and 38 patterns also coincide with the two conjectured dimer models of A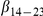 at pH 8.4 based on electron microscopy observation, ThT binding and molecular mechanics energy minimizations [25].

Overall, we see that the three most populated antiparallel A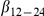 registries (i+j = 37,38 and 39) determined at pH 2.4 and 5.0 coincide with the registries observed for the fibrils of A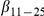 and A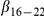 at acidic and neutral pH, with the 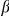-strands segments spanning the CHC region. The presence of out-of-register 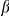-sheets indicate that perfect alignment of the CHC region is not an absolute requirement. On the other hand, the most populated antiparallel registers at pH 8.4 include an unexpected 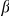-strand segments spanning Val12-Lys16 and the i+j = 37 registry.

#### Potential of mean force of dimerization

With the extensive number of configurations available, which goes beyond the data obtained by umbrella sampling simulations, we have constructed the potential of mean force (PMF) as a function of the interchain distance, which is the center of mass distance between the heavy atoms of the two CHC regions. The PMF was constructed as follows. A one-dimensional grid with respect to the interchain distance was created to account for the number of sampled conformations in each grid, denoted as Ni. The relative conformational free energy, Vi, was calculated as 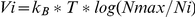, where T is the absolute temperature, 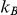 is the Boltzmann constant and Nmax is the largest number of sampled conformations counted in one bin.

**Figure 8:**
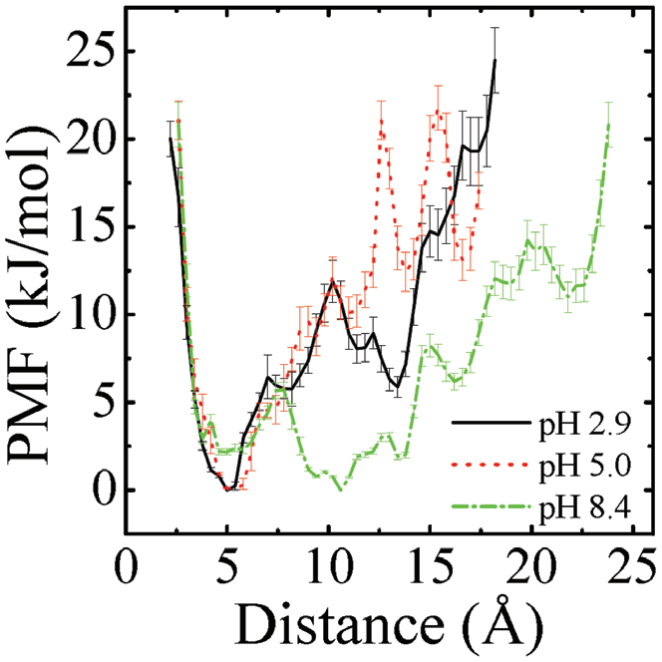
Potential of mean force (in kJ/mol) as a function of the inter-chain distance which is between the center of mass (COM) of the heavy atoms of two hydrophobic patches (A 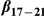
 **).** The statistical errors are below 2 kJ/mol.

Fig. 8 shows the PMFs for the three pH values. The PMF at pH 2.9 (solid line) has two main basins of attraction: the global minimum is located around the interchain distance of 5 Å; the other, 6 kJ/mol above, sits near 13 Å. The separating barrier between the two states is about 11.9
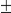
1.2 kJ/mol. For the PMF at pH 5.0 (dot line), the global minimum is still around 5 Å. The second minimum is located 12 Å and has a higher free energy of 10 kJ/mol. The barrier between the two minima is 12.2
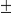
1.0 kJ/mol. This similarity in the PMF profiles makes it clear that our 1D reaction coordinate does not enable us to distinguish dimerization at pH 2.9 and 5.0. In contrast, for the PMF at pH 8.4 (dashed dot line), the global minimum is located at 11 Å, vs. 5 Å for pH 2.9 and 5.0. The overall profile of the PMF at pH 8.4 is much broader than those at pH 2.9 and 5.0, indicating that the CHC residues are much less organized and interacting at pH 8.4 than at pH 2.9 and 5.0.

#### Secondary structure preference

**Figure 9:**
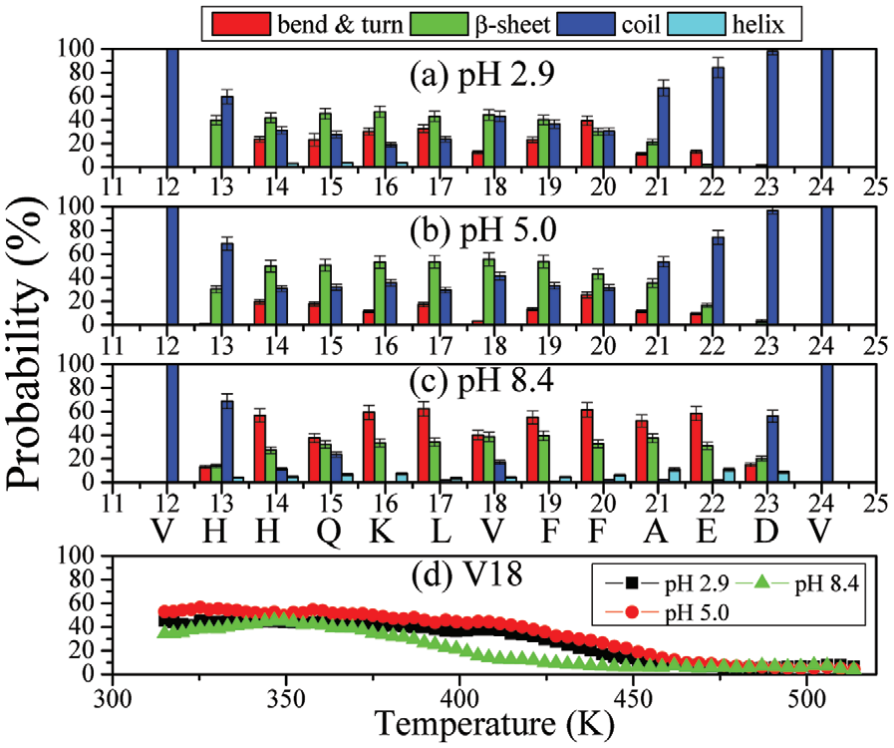
Secondary structure preference for each residue at T = 315K. The statistical errors are below 3% (a,b and c). The 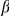 structure propensity of the middle residue, V18, as a function of temperature(d).

To further investigate the pH effects on the configurations, we have calculated the ensemble-averaged secondary structure preferences of each residue at 315 K as shown on Fig. 9a–9c. The secondary structures calculated by the DSSP algorithm are divided into four classes [47]: 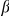-sheet, bend/turn, helix and coil. It is noted that the secondary structure profiles at pH 2.8 and 5.0 are very similar and differ markedly from that at pH 8.4. While bend-turn dominates over 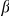-strand from His14 to Glu22 at pH 8.4, at lower pH the residues 14–20 and thus the CHC region have a preference for 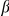-strand, and then coil and turn-bend. At pH 2.9 and 5.0, the probability for the 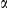-helical conformation is very low. In contrast, at pH 8.4 the C-terminal segment Phe19-Glu23 has a discernible population (10%) for helical secondary structure. The temperature dependence of the secondary structure is illustrated in Fig. 9d where the 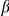 structure propensities of the middle residue, Val18, are shown as a function of pH. Under nearly all temperatures, the 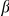 structure propensities at pH 5.0 are larger than those at pH 2.9 and pH 8.4 with that at pH 8.4 being the smallest. In addition, the propensity does not change much within 315–330 K, indicating that the difference may be very small at the experimental temperature of 296 K.

The Val-COOH pKa1 is 2.29 and Val-Amino pKa2 is 9.74 [56]. Within the pH range 2.9 to 8.4 the charge state of the peptide in the experiment is zwitterionic, NH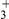 and COO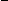 for N and C terminus, respectively. Thus the experimental observed pH-dependent aggregation behaviors have little contributions from the peptide termini. We used the ACE and NME as capping on the N and C terminus, respectively. The simulation data show that both termini are very flexible and their secondary structures (Val12 and Val24) are dominantly random coil. The capping groups, either neutral groups or zwitterionic ones, may not have significant effects on peptide conformation itself. For the peptide dimer aggregation, none of the nine dominant dimerization patterns found (Fig. 7) has large contributions from the terminus-terminus interactions which may also resort to the highly flexible nature of both termini. Therefore, we think the capping groups in this study have little effects on the overall conformations of the peptide aggregates in the very early aggregation process and in the final products. This is further supported by the experimental results of Bu et al. [57] which showed that A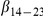 with acetyl N-terminus and NH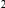 C-terminus forms fibrils at pH 3 and our finding that A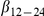 in its zwitterion also forms fibrils at low pH.

Overall, when the pH varies from 5 to 8.4 and only the histidine residues change from the charge state of +1 to 0, the secondary structures, nevertheless, change drastically. This 2D structure modification results from a different balance between intra-peptide and inter-peptide interactions, but the contribution of peptide-water interactions remains to be determined.

#### pH effects on hydration

**Figure 10:**
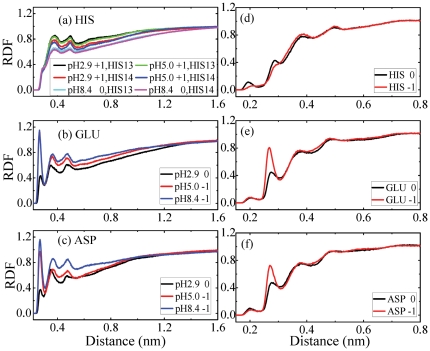
The radial distribution functions of water molecules around neutral/charged residues within A 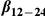
 **peptides.** (a) HIS, (b) GLU and (c) ASP. The statistical errors are below 1%. The radial distribution functions of water molecules around isolated neutral/charged residues: (d) HIS, (e) GLU and (f) ASP. The statistical errors are below 0.5%.

The protonation states of His13, His14, Glu22 and Asp23 vary with pH. The radial distribution functions (RDFs) between residues (only heavy atoms were considered) and water molecules (only oxygen atoms were considered) are plotted in Fig. 10a–c. The RDF gives the ensemble averaged number (or density) of solute-oxygen water pairs found at a distance 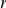.

In the context of the dimer, the height of the first solvation peak (1.2 unit) for the charged residues Glu and Asp is higher than that of their neutral species (0.4 unit), and the first peaks for the negatively charged Glu and Asp residues are much higher than the first peaks for the positively charged His residues. Note the RDFs for the two His13 and His14 residues are almost indistinguishable (Fig. 10a).

To decouple the influence of nearby residues and dimerization we calculated the RDFs of the isolated His, Asp and Glu residues blocked by ACE and NME. The resulting RDFs are shown in Fig. 10 d–f. Interestingly, the hydration structures of the isolated residues are rather similar to those in the context of A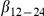, though the height of the first peak for the neutral Glu residue is not as high.

Overall, it is clear from panels 10a–c that the interactions between water molecules and the Glu, Asp and His residues cannot explain the differences in the aggregation properties of the dimer upon varying from pH 5.0 to 8.4. There is, however, a significant difference in the heights of the first peak for the negatively Glu and the neutral Glu residues which surely impacts the free energy surfaces from pH 2.9 to 5.0.

## Discussion

The initial self-assembly of polypeptides leading to the formation of 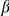-sheet structure is a critical step in the amyloid formation, providing templates for the further amyloid growth. We have conducted extensive REMD studies of dimerization of A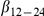 at different pHs and showed that the high propensity to form 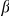-sheet containing dimers is linked directly to the consequent fast amyloid self-assembly. Specifically, the dimerization is strongly regulated by pH (5.0
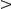
2.9
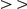
8.4) due to modulation of the electrostatic attraction between the side-chains of His13/His14 and Glu22/Asp23 and if they are positively and negatively charged, respectively, as happened at pH 5.0, this favors the antiparallel alignment and stable 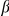-sheet patten as assessed in terms of a number of inter-peptide hydrogen bonds and their energy (Fig. 3 and 5). Employing constant pH molecular dynamics simulations of two A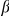 peptide segments, A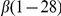 and A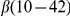, Khandogin and Brooks III also found that the folding landscape of the peptides is strongly modulated by pH and is most favorable for hydrophobically driven aggregation at pH 6 [58].

The dimerization is a key event in the amyloid cascade as the A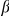 dimers can be converted into stable synaptotoxic protofibrils [59]. Recently it has been shown that the A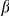 dimers are the most abundant form of soluble oligomers detectable in the cortices regions of the brain from typical Alzheimer's disease subjects and at subnanomolar concentrations they induce hyperphosphorylation of tau in neurons and disrupt the microtubule cytoskeleton, causing neuritic degeneration [60]. The regulation of the brain pH as well as the generation of activity-related pH changes and their functional consequences is the subject of extensive research [61]. The studies of pH in the CNS is distinguished by the occurrence of rapid increases or decreases in pH values that arise from electrical activity. These changes take place in time frames from milliseconds to minutes, involving neurons as well as glia, and occur in both the intracellular and extracellular compartments [62]–[65]. It was also suggested that mild acidification of the intracellular compartments such as endosomes as well as acidic environment of lysosomes can favors amyloidogenesis [66], [67]. Consequently, the populations of transient oligomers [68] as well as the amyloid fibril morphology and kinetics [15], [69]–[71] depend on pH.

In this study, we related the pH-dependent dimerization of A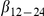 with its subsequent amyloid growth monitored by AFM and ThT fluorescence and showed that the formation of stable 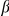-sheet dimers is critical in the amyloid formation, i.e. the length of 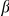-sheet in the dimer is 13, 14.3 and 9 at pH 2.9, 5.0 and 8.4, respectively, and thus largest at pH 5.0.

Our experimental results at 296 K show that the amyloid self-assembly proceeded very rapidly at pH 5.0 leading to the formation of a dense network of amyloid fibrils. At pH 2.9 amyloid fibrils were shorter, thinner and in a less quantity than at pH 5.0 (Fig. 1 a–c). At pH 8.4 the amyloid formation was significantly depressed as evident from both low value of thioflavin T fluorescence and AFM imaging. Thus, pH 5.0 proved to be the most and pH 8.4 the least amyloid-prone conditions in our experiments.

In good agreement with these, the dimer conformations at pH 8.4 and 315 K are predicted to be very inhomogeneous and in dynamic equilibrium with many states, as revealed by its free energy surface (Fig. 4), its higher turn-bend secondary structure composition and its more diffuse Sc-Sc contact map probability (Fig. 5). Analysis of the most populated and extended anti-parallel 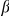 sheet registries of the dimers at three pH values (Fig. 6 and 7) and the potential of mean force with the global minimum at 11 Å vs 5 Å at pH 8.4 and pH 5.0; 2.9, respectively, further explains why A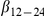 does not form amyloid fibrils at pH 8.4. It is important to note that the temperature difference between our experiments (296 K) and simulations (315 K) is rather small. Based on our previous REMD simulation study [34] and the temperature-dependent 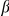 structure propensities shown in Fig. 9d, the computational results at 315 K will not change much from those at 296 K.

In contrast to the simulations at pH 8.4, the simulations at pH 2.9 and 5.0 at 315 K lead to more defined free energy surfaces and Sc-Sc contact maps with various antiparallel 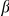-sheets and disordered states (Fig. 4–7). In both conditions, the amorphous states are more populated than the anti-parallel stranded geometries that match the H-bond registers found by NMR-solid state experiment for A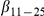 and A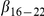 fibrils at acidic and neutral pH [19], [18]. This indicates that the A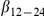 dimer does not necessarily encode by itself the final register of the fibrils, consistent with the previous PMF calculation on the A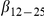 dimer [20].

Examining the balance between peptide-peptide and peptide-water interactions, we provide additional physical rationale to the A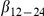 pH-dependent behaviors. At pH 5.0, the hydrophobic interactions between the two CHC regions are further stabilized by the strong salt-bridges between His(+1) and Asp(−1)/Glu(−1) residues, whereas at pH 2.9 and 8.4, these favorable inter-chain salt-bridge interactions are lost which results in the longest 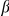-sheet at pH 5.0. Comparing the case at pH 8.4 to that at pH 2.9, there is a significant difference in the heights of the first RDF peak for the negatively Glu (at pH 8.4) and the neutral Glu residues (at pH 2.9), indicating that both peptide-water and peptide-peptide interactions contribute to the lower 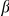-sheet content of the A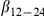 dimer at pH 8.4 than that at pH 2.9 (Fig. 10). The critical role of the charges of His13 and His14 residues found in the A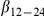 fibril formation is consistent with a previous experimental study on A
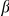
40 fibrillation with His13 and His14 replaced by Gln where fibrillogenesis were significantly retarded [72]. This finding is particularly intriguing because His13 and His14 residues are binding sites for metal ions Cu and Zn, which have a dramatic impact on A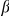 aggregation behaviors and account for the mechanism of reactive oxygen species [73].

Thus, the analysis at atomic resolution of the self-assembly of the A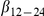 segment corresponding to the hydrophobic core of full length A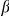 peptide provides an insight into the early events of aggregation and specific hydrogen bond patterns and side-chain interactions triggering this process. Understanding the interplay between molecular interactions involved in amyloid assembly provides the rational basis for developing protective strategies and inhibiting this harmful process.
